# Supplementary material for: Shotgun Label-Free Proteomic Analysis for Identification of a Potential Diagnostic Biomarker for Pancreatic Cancer
Source: Biomedicines. 2025 Oct 27;13(11):2631. doi: 10.3390/biomedicines13112631 (PMC12649963; doi:10.3390/biomedicines13112631)
Supplement: Supplementary file 1 [file biomedicines-13-02631-s001.zip › Supplementary table.pdf]

Table S1. Differentially expressed proteins in BxPC-3 cells.

No.	ID	Protein name	Number of amino acids	Spectral counting		
				HPNE	BxPC-3	Fold chang (Rsc)
1	FINC_HUMAN	Fibronectin	2386	109	1	-5.814
2	TAGL_HUMAN	Transgelin	201	38	0	-5.156
3	TPM2_HUMAN	Tropomyosin beta chain	284	29	0	-4.778
4	PAI1_HUMAN	Plasminogen activator inhibitor	402	20	0	-4.267
5	ANXA6_HUMAN	Annexin A6	673	16	0	-3.965
6	EF1A3_HUMAN	Putative elongation factor 1-alpha-like 3	462	15	0	-3.878
7	CALD1_HUMAN	Caldesmon	793	14	0	-3.787
8	VIME_HUMAN	Vimentin	466	180	16	-3.607
9	CKAP4_HUMAN	Cytoskeleton-associated protein 4	602	10	0	-3.347
10	CO1A1_HUMAN	Collagen alpha-1(I) chain	1464	10	0	-3.347
11	TLN1_HUMAN	Talin-1	2541	9	0	-3.212
12	RAB1B_HUMAN	Ras-related protein Rab-1B	201	8	0	-3.064
13	RTN4_HUMAN	Reticulon-4	1192	8	0	-3.064
14	PGAM2_HUMAN	Phosphoglycerate mutase 2	253	8	0	-3.064
15	TPM4_HUMAN	Tropomyosin alpha-4 chain	248	36	4	-3.009
16	RAB1A_HUMAN	Ras-related protein Rab-1A	205	7	0	-2.899
17	H2B1J_HUMAN	Histone H2B type 1-J	126	7	0	-2.899
18	MT1G_HUMAN	Metallothionein-1G	62	6	0	-2.712
19	CBR1_HUMAN	Carbonyl reductase [NADPH] 1	277	6	0	-2.712
20	MT2_HUMAN	Metallothionein-2	61	6	0	-2.712
21	STMN2_HUMAN	Stathmin-2	179	6	0	-2.712
22	AINX_HUMAN	Alpha-internexin	499	5	0	-2.498
23	PLIN3_HUMAN	Perilipin-3	434	5	0	-2.498
24	ARF3_HUMAN	ADP-ribosylation factor 3	181	5	0	-2.498

25	RL37A_HUMAN	60S ribosomal protein L37a	92	4	0	-2.246
26	RL19_HUMAN	60S ribosomal protein L19	196	4	0	-2.246
27	HSPB6_HUMAN	Heat shock protein beta-6	160	4	0	-2.246
28	CALU_HUMAN	Calumenin	315	4	0	-2.246
29	GNAI2_HUMAN	Guanine nucleotide-binding protein G(i) subunit alpha-2	355	4	0	-2.246
30	RL35_HUMAN	60S ribosomal protein L35	123	4	0	-2.246
31	WDR1_HUMAN	WD repeat-containing protein 1	606	8	1	-2.216
32	AHNK_HUMAN	Neuroblast differentiation-associated protein	5890	34	8	-2.111
33	GDIR1_HUMAN	Rho GDP-dissociation inhibitor 1	204	7	1	-2.050
34	CO6A3_HUMAN	Collagen alpha-3(VI) chain	3177	3	0	-1.941
35	CBR3_HUMAN	Carbonyl reductase [NADPH] 3	277	3	0	-1.941
36	ACLY_HUMAN	ATP-citrate synthase	1101	3	0	-1.941
37	SC22B_HUMAN	Vesicle-trafficking protein SEC22b	215	3	0	-1.941
38	RAB5C_HUMAN	Ras-related protein Rab-5C	216	3	0	-1.941
39	COR1C_HUMAN	Coronin-1C	474	3	0	-1.941
40	UCHL1_HUMAN	Ubiquitin carboxyl-terminal hydrolase isozyme L1	223	3	0	-1.941
41	SPI2_HUMAN	Serpin I2	405	3	0	-1.941
42	TRI10_HUMAN	Tripartite motif-containing protein 10	481	3	0	-1.941
43	RL18A_HUMAN	60S ribosomal protein L18a	176	3	0	-1.941
44	FBLN1_HUMAN	Fibulin-1	703	3	0	-1.941
45	ARF5_HUMAN	ADP-ribosylation factor 5	180	3	0	-1.941
46	MVP_HUMAN	Major vault protein	893	6	1	-1.864
47	RL24_HUMAN	60S ribosomal protein L24	157	6	1	-1.864
48	TPM1_HUMAN	Tropomyosin alpha-1 chain	284	25	7	-1.849
49	GDIB_HUMAN	Rab GDP dissociation inhibitor beta	445	9	2	-1.833
50	GELS_HUMAN	Gelsolin OS=Homo sapiens	782	9	2	-1.833
51	PRDX6_HUMAN	Peroxiredoxin-6	224	12	3	-1.817
52	PERI_HUMAN	Peripherin	470	15	4	-1.807
53	LASP1_HUMAN	LIM and SH3 domain protein 1	261	14	4	-1.715

54	PDIA6_HUMAN	Protein disulfide-isomerase A6	440	8	2	-1.685
55	RS7_HUMAN	40S ribosomal protein S7	194	5	1	-1.649
56	CAN2_HUMAN	Calpain-2 catalytic subunit	700	5	1	-1.649
57	LEG1_HUMAN	Galectin-1	135	38	13	-1.642
58	MYH14_HUMAN	Myosin-14	1995	21	7	-1.609
59	STMN1_HUMAN	Stathmin	149	10	3	-1.581
60	GRHL1_HUMAN	Grainyhead-like protein 1 homolog	618	2	0	-1.554
61	PTRF_HUMAN	Polymerase I and transcript release factor	390	2	0	-1.554
62	ARPC5_HUMAN	Actin-related protein 2/3 complex subunit 5	151	2	0	-1.554
63	RL31_HUMAN	60S ribosomal protein L31	125	2	0	-1.554
64	PYRG1_HUMAN	CTP synthase 1	591	2	0	-1.554
65	CSRP1_HUMAN	Cysteine and glycine-rich protein 1	193	2	0	-1.554
66	RRBP1_HUMAN	Ribosome-binding protein 1	1410	2	0	-1.554
67	MTAP2_HUMAN	Microtubule-associated protein 2	1827	2	0	-1.554
68	ECHB_HUMAN	Trifunctional enzyme subunit beta, mitochondrial	474	2	0	-1.554
69	RL9_HUMAN	60S ribosomal protein L9	192	2	0	-1.554
70	SMC5_HUMAN	Structural maintenance of chromosomes protein 5	1101	2	0	-1.554
71	MAP1B_HUMAN	Microtubule-associated protein 1B	2468	2	0	-1.554
72	CCD33_HUMAN	Coiled-coil domain-containing protein 33	958	2	0	-1.554
73	NNMT_HUMAN	Nicotinamide N-methyltransferase	264	2	0	-1.554
74	KRT82_HUMAN	Keratin, type II cuticular Hb2	513	2	0	-1.554
75	CHD4_HUMAN	Chromodomain-helicase-DNA-binding protein 4	1912	2	0	-1.554
76	XPP3_HUMAN	Probable Xaa-Pro aminopeptidase 3	507	2	0	-1.554
77	KATL2_HUMAN	Katanin p60 ATPase-containing subunit A-like 2	538	2	0	-1.554
78	GNAI1_HUMAN	Guanine nucleotide-binding protein G(i) subunit alpha-1	354	2	0	-1.554
79	FKB10_HUMAN	Peptidyl-prolyl cis-trans isomerase FKBP10	582	2	0	-1.554
80	COPD_HUMAN	Coatomer subunit delta	511	2	0	-1.554
81	USO1_HUMAN	General vesicular transport factor p115	962	2	0	-1.554
82	SEPT7_HUMAN	Septin-7	437	2	0	-1.554

83	5NTD_HUMAN	5~-nucleotidase	574	2	0	-1.554
84	BCL7B_HUMAN	B-cell CLL/lymphoma 7 protein family member B	202	2	0	-1.554
85	CO3A1_HUMAN	Collagen alpha-1(III) chain	1466	2	0	-1.554
86	RS15_HUMAN	40S ribosomal protein S15	145	2	0	-1.554
87	IDHC_HUMAN	Isocitrate dehydrogenase [NADP] cytoplasmic	414	2	0	-1.554
88	IPO7_HUMAN	Importin-7	1038	2	0	-1.554
89	SYCP2_HUMAN	Synaptonemal complex protein 2	1530	2	0	-1.554
90	VIGLN_HUMAN	Vigilin	1268	2	0	-1.554
91	NSF1C_HUMAN	NSFL1 cofactor p47	370	2	0	-1.554
92	CNTRL_HUMAN	Centriolin	2325	2	0	-1.554
93	ARF1_HUMAN	ADP-ribosylation factor 1	181	7	2	-1.520
94	RS16_HUMAN	40S ribosomal protein S16	146	7	2	-1.520
95	DCD_HUMAN	Dermcidin	110	12	4	-1.512
96	PDIA1_HUMAN	Protein disulfide-isomerase	508	25	10	-1.401
97	MYH10_HUMAN	Myosin-10	1976	18	7	-1.400
98	RSSA_HUMAN	40S ribosomal protein SA	295	11	4	-1.399
99	FAS_HUMAN	Fatty acid synthase	2511	11	4	-1.399
100	IMB1_HUMAN	Importin subunit beta-1	876	4	1	-1.398
101	GDIA_HUMAN	Rab GDP dissociation inhibitor alpha	447	4	1	-1.398
102	RS23_HUMAN	40S ribosomal protein S23	143	4	1	-1.398
103	MT1E_HUMAN	Metallothionein-1E	61	4	1	-1.398
104	RS8_HUMAN	40S ribosomal protein S8	208	4	1	-1.398
105	RS4Y1_HUMAN	40S ribosomal protein S4, Y isoform 1	263	4	1	-1.398
106	CLH1_HUMAN	Clathrin heavy chain 1	1675	13	5	-1.366
107	RAB10_HUMAN	Ras-related protein Rab-10	200	6	2	-1.333
108	CD44_HUMAN	CD44 antigen	742	6	2	-1.333
109	NDK8_HUMAN	Putative nucleoside diphosphate kinase	137	10	4	-1.276
110	RS18_HUMAN	40S ribosomal protein S18	152	10	4	-1.276
111	1433T_HUMAN	14-3-3 protein theta	245	12	5	-1.260

112	CALR_HUMAN	Calreticulin	417	14	6	-1.249
113	MYH11_HUMAN	Myosin-11	1972	22	10	-1.225
114	RS2_HUMAN	40S ribosomal protein S2	293	15	7	-1.155
115	RL8_HUMAN	60S ribosomal protein L8	257	7	3	-1.133
116	K1C27_HUMAN	Keratin, type I cytoskeletal 27	459	5	2	-1.119
117	ITB1_HUMAN	Integrin beta-1	798	5	2	-1.119
118	MYL1_HUMAN	Myosin light chain 1/3, skeletal muscle isoform	194	3	1	-1.093
119	CLH2_HUMAN	Clathrin heavy chain 2	1640	3	1	-1.093
120	RAB13_HUMAN	Ras-related protein Rab-13	203	3	1	-1.093
121	RAB12_HUMAN	Ras-related protein Rab-12	244	3	1	-1.093
122	MT1A_HUMAN	Metallothionein-1A	61	3	1	-1.093
123	IPO5_HUMAN	Importin-5	1097	3	1	-1.093
124	RL38_HUMAN	60S ribosomal protein L38	70	3	1	-1.093
125	H2B1H_HUMAN	Histone H2B type 1-H	126	18	9	-1.086
126	MYH9_HUMAN	Myosin-9	1960	125	67	-1.077
127	FLNC_HUMAN	Filamin-C	2725	27	14	-1.067
128	ARF4_HUMAN	ADP-ribosylation factor 4	180	14	7	-1.063
129	TBA1B_HUMAN	Tubulin alpha-1B chain	451	67	36	-1.056
130	ANR31_HUMAN	Putative ankyrin repeat domain-containing protein 31	1873	1	0	-1.023
131	TLN2_HUMAN	Talin-2	2542	1	0	-1.023
132	RL3L_HUMAN	60S ribosomal protein L3-like	407	1	0	-1.023
133	ATX10_HUMAN	Ataxin-10	475	1	0	-1.023
134	RCN3_HUMAN	Reticulocalbin-3	328	1	0	-1.023
135	RINI_HUMAN	Ribonuclease inhibitor	461	1	0	-1.023
136	LRC59_HUMAN	Leucine-rich repeat-containing protein 59	307	1	0	-1.023
137	NB5R3_HUMAN	NADH-cytochrome b5 reductase 3	301	1	0	-1.023
138	TNPO1_HUMAN	Transportin-1	898	1	0	-1.023
139	RL26L_HUMAN	60S ribosomal protein L26-like 1	145	1	0	-1.023
140	LIMS1_HUMAN	LIM and senescent cell antigen-like-containing domain protein 1	325	1	0	-1.023

141	STAU1_HUMAN	Double-stranded RNA-binding protein Staufen homolog 1	577	1	0	-1.023
142	C1TC_HUMAN	C-1-tetrahydrofolate synthase, cytoplasmic	935	1	0	-1.023
143	LRC47_HUMAN	Leucine-rich repeat-containing protein 47	583	1	0	-1.023
144	PHX2A_HUMAN	Paired mesoderm homeobox protein 2A	284	1	0	-1.023
145	VA0D1_HUMAN	V-type proton ATPase subunit d 1	351	1	0	-1.023
146	RSU1_HUMAN	Ras suppressor protein 1	277	1	0	-1.023
147	CLD11_HUMAN	Claudin-11	207	1	0	-1.023
148	JIP4_HUMAN	C-Jun-amino-terminal kinase-interacting protein 4	1321	1	0	-1.023
149	IMA5_HUMAN	Importin subunit alpha-5	538	1	0	-1.023
150	CENPV_HUMAN	Centromere protein V	275	1	0	-1.023
151	MCM3_HUMAN	DNA replication licensing factor MCM3	808	1	0	-1.023
152	CSK23_HUMAN	Casein kinase II subunit alpha 3	391	1	0	-1.023
153	LMF2_HUMAN	Lipase maturation factor 2	707	1	0	-1.023
154	RM35_HUMAN	39S ribosomal protein L35, mitochondrial	188	1	0	-1.023
155	TPPC3_HUMAN	Trafficking protein particle complex subunit 3	180	1	0	-1.023
156	NALP3_HUMAN	NACHT, LRR and PYD domains-containing protein 3	1036	1	0	-1.023
157	RBGPR_HUMAN	Rab3 GTPase-activating protein non-catalytic subunit	1393	1	0	-1.023
158	MPP7_HUMAN	MAGUK p55 subfamily member 7	576	1	0	-1.023
159	NSF_HUMAN	Vesicle-fusing ATPase	744	1	0	-1.023
160	RHG12_HUMAN	Rho GTPase-activating protein 12	846	1	0	-1.023
161	PLOD1_HUMAN	Procollagen-lysine,2-oxoglutarate 5-dioxygenase 1	727	1	0	-1.023
162	CHD6_HUMAN	Chromodomain-helicase-DNA-binding protein 6	2715	1	0	-1.023
163	DCTN2_HUMAN	Dynactin subunit 2	401	1	0	-1.023
164	IF4G2_HUMAN	Eukaryotic translation initiation factor 4 gamma 2	907	1	0	-1.023
165	SSBP3_HUMAN	Single-stranded DNA-binding protein 3	388	1	0	-1.023
166	PTPRA_HUMAN	Receptor-type tyrosine-protein phosphatase alpha	802	1	0	-1.023
167	RINT1_HUMAN	RAD50-interacting protein 1	792	1	0	-1.023
168	MARCS_HUMAN	Myristoylated alanine-rich C-kinase substrate	332	1	0	-1.023
169	BGAT_HUMAN	Histo-blood group ABO system transferase	354	1	0	-1.023

170	TMEDA_HUMAN	Transmembrane emp24 domain-containing protein 10	219	1	0	-1.023
171	GLGB_HUMAN	1,4-alpha-glucan-branching enzyme	702	1	0	-1.023
172	OR7D2_HUMAN	Olfactory receptor 7D2	312	1	0	-1.023
173	CREG2_HUMAN	Protein CREG2	290	1	0	-1.023
174	STK39_HUMAN	STE20/SPS1-related proline-alanine-rich protein kinase	545	1	0	-1.023
175	MED12_HUMAN	Mediator of RNA polymerase II transcription subunit 12	2177	1	0	-1.023
176	PSB7_HUMAN	Proteasome subunit beta type-7	277	1	0	-1.023
177	PCDH9_HUMAN	Protocadherin-9	1237	1	0	-1.023
178	MXRA8_HUMAN	Matrix-remodeling-associated protein 8	442	1	0	-1.023
179	LACTB_HUMAN	Serine beta-lactamase-like protein LACTB, mitochondrial	547	1	0	-1.023
180	VPP4_HUMAN	V-type proton ATPase 116 kDa subunit a isoform 4	840	1	0	-1.023
181	SLX4_HUMAN	Structure-specific endonuclease subunit SLX4	1834	1	0	-1.023
182	DDX1_HUMAN	ATP-dependent RNA helicase DDX1	740	1	0	-1.023
183	DHX9_HUMAN	ATP-dependent RNA helicase A	1270	1	0	-1.023
184	CD158_HUMAN	Coiled-coil domain-containing protein 158	1113	1	0	-1.023
185	CTR2_HUMAN	Cationic amino acid transporter 2	658	1	0	-1.023
186	AT2C1_HUMAN	Calcium-transporting ATPase type 2C member 1	919	1	0	-1.023
187	CX057_HUMAN	Uncharacterized protein CXorf57	855	1	0	-1.023
188	SSXT_HUMAN	Protein SSXT	418	1	0	-1.023
189	SH3G1_HUMAN	Endophilin-A2	368	1	0	-1.023
190	ACSL3_HUMAN	Long-chain-fatty-acid--CoA ligase 3	720	1	0	-1.023
191	CXCL2_HUMAN	C-X-C motif chemokine 2	107	1	0	-1.023
192	TTBK1_HUMAN	Tau-tubulin kinase 1	1321	1	0	-1.023
193	QCR2_HUMAN	Cytochrome b-c1 complex subunit 2, mitochondrial	453	1	0	-1.023
194	S14L1_HUMAN	SEC14-like protein 1	715	1	0	-1.023
195	RUVB1_HUMAN	RuvB-like 1	456	1	0	-1.023
196	SMCA4_HUMAN	Transcription activator BRG1	1647	1	0	-1.023
197	PRC2B_HUMAN	Protein PRRC2B	2229	1	0	-1.023
198	CGBP1_HUMAN	CGG triplet repeat-binding protein 1	167	1	0	-1.023

199	FBXL5_HUMAN	F-box/LRR-repeat protein 5	691	1	0	-1.023
200	TTC24_HUMAN	Tetratricopeptide repeat protein 24	582	1	0	-1.023
201	KR87P_HUMAN	Putative keratin-87 protein	255	1	0	-1.023
202	SC24D_HUMAN	Protein transport protein Sec24D	1032	1	0	-1.023
203	ARFG1_HUMAN	ADP-ribosylation factor GTPase-activating protein 1	406	1	0	-1.023
204	NXP20_HUMAN	Protein NOXP20	563	1	0	-1.023
205	GNA12_HUMAN	Guanine nucleotide-binding protein subunit alpha-12	381	1	0	-1.023
206	P4HA2_HUMAN	Prolyl 4-hydroxylase subunit alpha-2	535	1	0	-1.023
207	AP2B1_HUMAN	AP-2 complex subunit beta	937	1	0	-1.023
208	AMPL_HUMAN	Cytosol aminopeptidase	519	1	0	-1.023
209	PUR9_HUMAN	Bifunctional purine biosynthesis protein PURH	592	1	0	-1.023
210	PICAL_HUMAN	Phosphatidylinositol-binding clathrin assembly protein	652	1	0	-1.023
211	NEST_HUMAN	Nestin	1621	1	0	-1.023
212	TRPC4_HUMAN	Short transient receptor potential channel 4	977	1	0	-1.023
213	KIF5C_HUMAN	Kinesin heavy chain isoform 5C	957	1	0	-1.023
214	RS24_HUMAN	40S ribosomal protein S24	133	1	0	-1.023
215	ARPC3_HUMAN	Actin-related protein 2/3 complex subunit 3	178	1	0	-1.023
216	SPTB1_HUMAN	Spectrin beta chain, erythrocytic	2137	1	0	-1.023
217	ARHG9_HUMAN	Rho guanine nucleotide exchange factor 9	516	1	0	-1.023
218	ATP4A_HUMAN	Potassium-transporting ATPase alpha chain 1	1035	1	0	-1.023
219	CSPG4_HUMAN	Chondroitin sulfate proteoglycan 4	2322	1	0	-1.023
220	F10A5_HUMAN	Putative protein FAM10A5	369	1	0	-1.023
221	CO6A1_HUMAN	Collagen alpha-1(VI) chain	1028	1	0	-1.023
222	SPD2B_HUMAN	SH3 and PX domain-containing protein 2B	911	1	0	-1.023
223	DEK_HUMAN	Protein DEK	375	1	0	-1.023
224	CC033_HUMAN	Protein C3orf33	294	1	0	-1.023
225	FERM2_HUMAN	Fermitin family homolog 2	680	1	0	-1.023
226	MK15_HUMAN	Mitogen-activated protein kinase 15	544	1	0	-1.023
227	SUMO3_HUMAN	Small ubiquitin-related modifier 3	103	1	0	-1.023

228	MPCP_HUMAN	Phosphate carrier protein, mitochondrial	362	1	0	-1.023
229	CI050_HUMAN	Uncharacterized protein C9orf50	431	1	0	-1.023
230	CN080_HUMAN	Uncharacterized protein C14orf80	495	1	0	-1.023
231	EHD2_HUMAN	EH domain-containing protein 2	543	1	0	-1.023
232	CD166_HUMAN	CD166 antigen	583	1	0	-1.023
233	DEN1A_HUMAN	DENN domain-containing protein 1A	1009	1	0	-1.023
234	CUX1_HUMAN	Homeobox protein cut-like 1	1505	1	0	-1.023
235	CCD57_HUMAN	Coiled-coil domain-containing protein 57	916	1	0	-1.023
236	SRPR_HUMAN	Signal recognition particle receptor subunit alpha	638	1	0	-1.023
237	LA_HUMAN	Lupus La protein	408	1	0	-1.023
238	GASP1_HUMAN	G-protein coupled receptor-associated sorting protein 1	1395	1	0	-1.023
239	CRK_HUMAN	Adapter molecule crk	304	1	0	-1.023
240	MROH9_HUMAN	Maestro heat-like repeat-containing protein family member 9	573	1	0	-1.023
241	TBCA_HUMAN	Tubulin-specific chaperone A	108	1	0	-1.023
242	CCNC_HUMAN	Cyclin-C	283	1	0	-1.023
243	FEM1C_HUMAN	Protein fem-1 homolog C	617	1	0	-1.023
244	KDM4A_HUMAN	Lysine-specific demethylase 4A	1064	1	0	-1.023
245	FPPS_HUMAN	Farnesyl pyrophosphate synthase	419	1	0	-1.023
246	MK03_HUMAN	Mitogen-activated protein kinase 3	379	1	0	-1.023
247	THIC_HUMAN	Acetyl-CoA acetyltransferase, cytosolic	397	1	0	-1.023
248	THSD4_HUMAN	Thrombospondin type-1 domain-containing protein 4	1018	1	0	-1.023
249	MPRI_HUMAN	Cation-independent mannose-6-phosphate receptor	2491	1	0	-1.023
250	B2MG_HUMAN	Beta-2-microglobulin	119	1	0	-1.023
251	CASS4_HUMAN	Cas scaffolding protein family member 4	786	1	0	-1.023
252	UVSSA_HUMAN	UV-stimulated scaffold protein A	709	1	0	-1.023
253	XPO1_HUMAN	Exportin-1	1071	1	0	-1.023
254	NU155_HUMAN	Nuclear pore complex protein Nup155	1391	1	0	-1.023
255	HSP74_HUMAN	Heat shock 70 kDa protein 4	840	1	0	-1.023
256	CP2U1_HUMAN	Cytochrome P450 2U1	544	1	0	-1.023

257	SYYC_HUMAN	Tyrosine--tRNA ligase, cytoplasmic	528	1	0	-1.023
258	CYFP2_HUMAN	Cytoplasmic FMR1-interacting protein 2	1278	1	0	-1.023
259	KAP3_HUMAN	cAMP-dependent protein kinase type II-beta regulatory subunit	418	1	0	-1.023
260	CABP1_HUMAN	Calcium-binding protein 1	370	1	0	-1.023
261	SCAM4_HUMAN	Secretory carrier-associated membrane protein 4	229	1	0	-1.023
262	DHE4_HUMAN	Glutamate dehydrogenase 2, mitochondrial	558	1	0	-1.023
263	CENPF_HUMAN	Centromere protein F	3210	1	0	-1.023
264	AURKA_HUMAN	Aurora kinase A	403	1	0	-1.023
265	SEPT8_HUMAN	Septin-8	483	1	0	-1.023
266	PCM1_HUMAN	Pericentriolar material 1 protein	2024	1	0	-1.023
267	DEDD_HUMAN	Death effector domain-containing protein	318	1	0	-1.023
268	IMA7_HUMAN	Importin subunit alpha-7	536	1	0	-1.023
269	VDAC3_HUMAN	Voltage-dependent anion-selective channel protein 3	283	1	0	-1.023
270	SRP14_HUMAN	Signal recognition particle 14 kDa protein	136	1	0	-1.023
271	MT1M_HUMAN	Metallothionein-1M	61	1	0	-1.023
272	MT1B_HUMAN	Metallothionein-1B	61	1	0	-1.023
273	PYGB_HUMAN	Glycogen phosphorylase, brain form	843	1	0	-1.023
274	NEDD8_HUMAN	NEDD8	81	1	0	-1.023
275	COPG1_HUMAN	Coatomer subunit gamma-1	874	1	0	-1.023
276	NDEL1_HUMAN	Nuclear distribution protein nudE-like 1	345	1	0	-1.023
277	RSMB_HUMAN	Small nuclear ribonucleoprotein-associated proteins B and B~	240	1	0	-1.023
278	RALA_HUMAN	Ras-related protein Ral-A	206	1	0	-1.023
279	NEUL4_HUMAN	Neuralized-like protein 4	1562	1	0	-1.023
280	DHAK_HUMAN	Bifunctional ATP-dependent dihydroxyacetone kinase/FAD-AMP lyase (cyclizing)	575	1	0	-1.023
281	6PGD_HUMAN	6-phosphogluconate dehydrogenase, decarboxylating	483	1	0	-1.023
282	CYR61_HUMAN	Protein CYR61	381	1	0	-1.023
283	CS010_HUMAN	UPF0556 protein C19orf10	173	1	0	-1.023
284	SAP18_HUMAN	Histone deacetylase complex subunit SAP18	153	1	0	-1.023
285	CC147_HUMAN	Coiled-coil domain-containing protein 147	872	1	0	-1.023

286	UGGG1_HUMAN	UDP-glucose:glycoprotein glucosyltransferase 1	1555	1	0	-1.023
287	COX2_HUMAN	Cytochrome c oxidase subunit 2	227	1	0	-1.023
288	M3K15_HUMAN	Mitogen-activated protein kinase kinase kinase 15	1313	1	0	-1.023
289	H12_HUMAN	Histone H1.2	213	1	0	-1.023
290	ACSA_HUMAN	Acetyl-coenzyme A synthetase, cytoplasmic	701	1	0	-1.023
291	ZN474_HUMAN	Zinc finger protein 474	364	1	0	-1.023
292	CA167_HUMAN	Uncharacterized protein C1orf167	1468	1	0	-1.023
293	CXA1_HUMAN	Gap junction alpha-1 protein	382	1	0	-1.023
294	NEBL_HUMAN	Nebulette	1014	1	0	-1.023
295	GPKOW_HUMAN	G patch domain and KOW motifs-containing protein	476	1	0	-1.023
296	MAP4_HUMAN	Microtubule-associated protein 4	1152	1	0	-1.023
297	VGFR1_HUMAN	Vascular endothelial growth factor receptor 1	1338	1	0	-1.023
298	FXL19_HUMAN	F-box/LRR-repeat protein 19	694	1	0	-1.023
299	PSB1_HUMAN	Proteasome subunit beta type-1	241	1	0	-1.023
300	PPR1C_HUMAN	Protein phosphatase 1 regulatory subunit 1C	109	1	0	-1.023
301	S2547_HUMAN	Solute carrier family 25 member 47	308	1	0	-1.023
302	RAC2_HUMAN	Ras-related C3 botulinum toxin substrate 2	192	1	0	-1.023
303	ARC1B_HUMAN	Actin-related protein 2/3 complex subunit 1B	372	1	0	-1.023
304	NIBL1_HUMAN	Niban-like protein 1	746	1	0	-1.023
305	SRRM4_HUMAN	Serine/arginine repetitive matrix protein 4	611	1	0	-1.023
306	KMT2A_HUMAN	Histone-lysine N-methyltransferase 2A	3969	1	0	-1.023
307	RBM45_HUMAN	RNA-binding protein 45	476	1	0	-1.023
308	UB2V1_HUMAN	Ubiquitin-conjugating enzyme E2 variant 1	147	1	0	-1.023
309	COMD3_HUMAN	COMM domain-containing protein 3	195	1	0	-1.023
310	TMM47_HUMAN	Transmembrane protein 47	181	1	0	-1.023
311	CD151_HUMAN	CD151 antigen	253	1	0	-1.023
312	CC173_HUMAN	Coiled-coil domain-containing protein 173	552	1	0	-1.023
313	DPYL2_HUMAN	Dihydropyrimidinase-related protein 2	572	1	0	-1.023
314	ACADS_HUMAN	Short-chain specific acyl-CoA dehydrogenase, mitochondrial	412	1	0	-1.023

315	MKS1_HUMAN	Meckel syndrome type 1 protein	559	1	0	-1.023
316	NPT3_HUMAN	Sodium-dependent phosphate transport protein 3	439	1	0	-1.023
317	ANKE1_HUMAN	Ankyrin repeat and EF-hand domain-containing protein 1	776	1	0	-1.023
318	INSRR_HUMAN	Insulin receptor-related protein	1297	1	0	-1.023
319	CNGA2_HUMAN	Cyclic nucleotide-gated olfactory channel	664	1	0	-1.023
320	UGPA_HUMAN	UTP--glucose-1-phosphate uridylyltransferase	508	1	0	-1.023
321	PDLI7_HUMAN	PDZ and LIM domain protein 71	457	1	0	-1.023
322	GCP3_HUMAN	Gamma-tubulin complex component 3	907	1	0	-1.023
323	MAN1_HUMAN	Inner nuclear membrane protein Man1	911	1	0	-1.023
324	TLDC1_HUMAN	TLD domain-containing protein 1	456	1	0	-1.023
325	AP1B1_HUMAN	AP-1 complex subunit beta-1	949	1	0	-1.023
326	SPEE_HUMAN	Spermidine synthase	302	1	0	-1.023
327	AP2A1_HUMAN	AP-2 complex subunit alpha-1	977	1	0	-1.023
328	GMPPA_HUMAN	Mannose-1-phosphate guanyltransferase alpha	420	1	0	-1.023
329	MSTRO_HUMAN	Protein maestro	248	1	0	-1.023
330	PCDG6_HUMAN	Protocadherin gamma-A6	932	1	0	-1.023
331	K2C80_HUMAN	Keratin, type II cytoskeletal 80	452	1	4	1.048
332	HNRPF_HUMAN	Heterogeneous nuclear ribonucleoprotein F	415	1	4	1.048
333	RL22_HUMAN	60S ribosomal protein L22	128	1	4	1.048
334	PA2G4_HUMAN	Proliferation-associated protein 2G4	394	1	4	1.048
335	COX5A_HUMAN	Cytochrome c oxidase subunit 5A, mitochondrial	150	1	4	1.048
336	CTND1_HUMAN	Catenin delta-1	968	1	4	1.048
337	PLST_HUMAN	Plastin-3	630	4	11	1.049
338	H2BFS_HUMAN	Histone H2B type F-S	126	8	21	1.094
339	EZRI_HUMAN	Ezrin	586	15	38	1.102
340	CH60_HUMAN	60 kDa heat shock protein, mitochondrial	573	15	38	1.102
341	K2C6B_HUMAN	Keratin, type II cytoskeletal 6B	564	30	77	1.159
342	MDHM_HUMAN	Malate dehydrogenase, mitochondrial	338	2	7	1.170
343	H2A2B_HUMAN	Histone H2A type 2-B	130	2	7	1.170

344	ROA1_HUMAN	Heterogeneous nuclear ribonucleoprotein A1	372	2	7	1.170
345	HMGB1_HUMAN	High mobility group protein B1	215	2	7	1.170
346	K2C6A_HUMAN	Keratin, type II cytoskeletal 6A	564	33	86	1.185
347	CBX1_HUMAN	Chromobox protein homolog 1	185	0	2	1.204
348	IMA6_HUMAN	Importin subunit alpha-6	536	0	2	1.204
349	GLYM_HUMAN	Serine hydroxymethyltransferase, mitochondrial	504	0	2	1.204
350	HGB1A_HUMAN	Putative high mobility group protein B1-like 1	211	0	2	1.204
351	DX39B_HUMAN	Spliceosome RNA helicase DDX39B	428	0	2	1.204
352	CTNB1_HUMAN	Catenin beta-1	781	0	2	1.204
353	PLAK_HUMAN	Junction plakoglobin	745	0	2	1.204
354	TYB4_HUMAN	Thymosin beta-4	44	0	2	1.204
355	AL3A1_HUMAN	Aldehyde dehydrogenase, dimeric NADP-preferring	453	0	2	1.204
356	SEPT9_HUMAN	Septin-9	586	0	2	1.204
357	PSB2_HUMAN	Proteasome subunit beta type-2	201	0	2	1.204
358	HN1_HUMAN	Hematological and neurological expressed 1 protein	154	0	2	1.204
359	SETLP_HUMAN	Protein SETSIP	302	0	2	1.204
360	SMD1_HUMAN	Small nuclear ribonucleoprotein Sm D1	119	0	2	1.204
361	ARAP1_HUMAN	Arf-GAP with Rho-GAP domain, ANK repeat and PH domain-containing protein 1	1450	0	2	1.204
362	E2AK2_HUMAN	Interferon-induced, double-stranded RNA-activated protein kinase	551	0	2	1.204
363	O2T35_HUMAN	Olfactory receptor 2T35	323	0	2	1.204
364	SRSF4_HUMAN	Serine/arginine-rich splicing factor 4	494	0	2	1.204
365	AK1A1_HUMAN	Alcohol dehydrogenase [NADP(+)]	325	0	2	1.204
366	PSA1_HUMAN	Proteasome subunit alpha type-1	263	0	2	1.204
367	AQP3_HUMAN	Aquaporin-3	292	0	2	1.204
368	NTF2_HUMAN	Nuclear transport factor 2	127	0	2	1.204
369	HNRPL_HUMAN	Heterogeneous nuclear ribonucleoprotein L	589	0	2	1.204
370	AT1B3_HUMAN	Sodium/potassium-transporting ATPase subunit beta-3	279	0	2	1.204
371	SIX5_HUMAN	Homeobox protein SIX5	739	0	2	1.204
372	FUS_HUMAN	RNA-binding protein FUS	526	0	2	1.204

373	RB11B_HUMAN	Ras-related protein Rab-11B	218	0	2	1.204
374	PABP1_HUMAN	Polyadenylate-binding protein 1	636	0	2	1.204
375	HS105_HUMAN	Heat shock protein 105 kDa	858	0	2	1.204
376	AT1A1_HUMAN	Sodium/potassium-transporting ATPase subunit alpha-1	1023	0	2	1.204
377	BAF_HUMAN	Barrier-to-autointegration factor	89	0	2	1.204
378	ZMYM4_HUMAN	Zinc finger MYM-type protein 4	1548	0	2	1.204
379	RAC1_HUMAN	Ras-related C3 botulinum toxin substrate 1	192	0	2	1.204
380	MYO7A_HUMAN	Unconventional myosin-VIIa	2215	0	2	1.204
381	PSA5_HUMAN	Proteasome subunit alpha type-5	241	0	2	1.204
382	LAMP2_HUMAN	Lysosome-associated membrane glycoprotein 2	410	0	2	1.204
383	IMUP_HUMAN	Immortalization up-regulated protein	106	0	2	1.204
384	EMSA1_HUMAN	ELM2 and SANT domain-containing protein 1	1045	0	2	1.204
385	GCN1L_HUMAN	Translational activator GCN1	2671	0	2	1.204
386	HYOU1_HUMAN	Hypoxia up-regulated protein 1	999	0	2	1.204
387	IF4B_HUMAN	Eukaryotic translation initiation factor 4B	611	0	2	1.204
388	TRIM1_HUMAN	Probable E3 ubiquitin-protein ligase MID2	735	0	2	1.204
389	G3BP1_HUMAN	Ras GTPase-activating protein-binding protein 1	466	0	2	1.204
390	SYNE3_HUMAN	Nesprin-3	975	0	2	1.204
391	ROA3_HUMAN	Heterogeneous nuclear ribonucleoprotein A3	378	0	2	1.204
392	HNRPR_HUMAN	Heterogeneous nuclear ribonucleoprotein R	633	0	2	1.204
393	ANX11_HUMAN	Annexin A11	505	0	2	1.204
394	FKBP4_HUMAN	Peptidyl-prolyl cis-trans isomerase FKBP4	459	0	2	1.204
395	HS74L_HUMAN	Heat shock 70 kDa protein 4L	839	0	2	1.204
396	TCP4_HUMAN	Activated RNA polymerase II transcriptional coactivator p15	127	0	2	1.204
397	GSTP1_HUMAN	Glutathione S-transferase P	210	5	15	1.206
398	K2C75_HUMAN	Keratin, type II cytoskeletal 75	551	14	39	1.230
399	FLNB_HUMAN	Filamin-B	2602	11	32	1.270
400	NQO1_HUMAN	NAD(P)H dehydrogenase [quinone] 1	274	1	5	1.300
401	PSA6_HUMAN	Proteasome subunit alpha type-6	246	1	5	1.300

402	TXND5_HUMAN	Thioredoxin domain-containing protein 5	432	1	5	1.300
403	H90B4_HUMAN	Putative heat shock protein HSP 90-beta 4	505	1	5	1.300
404	SPTN4_HUMAN	Spectrin beta chain, non-erythrocytic 4	2564	2	8	1.336
405	RL21_HUMAN	60S ribosomal protein L21	160	2	8	1.336
406	K2C6C_HUMAN	Keratin, type II cytoskeletal 6C	564	28	82	1.345
407	SAHH_HUMAN	Adenosylhomocysteinase	432	3	11	1.354
408	HNRPM_HUMAN	Heterogeneous nuclear ribonucleoprotein M	730	9	30	1.438
409	RS27A_HUMAN	Ubiquitin-40S ribosomal protein S27a	156	1	6	1.514
410	TEBP_HUMAN	Prostaglandin E synthase 3	160	1	6	1.514
411	KRT85_HUMAN	Keratin, type II cuticular Hb5	507	1	6	1.514
412	K1C13_HUMAN	Keratin, type I cytoskeletal 13	458	14	48	1.523
413	K1C40_HUMAN	Keratin, type I cytoskeletal 40	431	0	3	1.592
414	CROCC_HUMAN	Rootletin	2017	0	3	1.592
415	ANXA8_HUMAN	Annexin A8	327	0	3	1.592
416	CAZA1_HUMAN	F-actin-capping protein subunit alpha-1	286	0	3	1.592
417	PTBP1_HUMAN	Polypyrimidine tract-binding protein 1	531	0	3	1.592
418	FKB1A_HUMAN	Peptidyl-prolyl cis-trans isomerase FKBP1A	108	0	3	1.592
419	PRDX5_HUMAN	Peroxiredoxin-5, mitochondrial	214	0	3	1.592
420	MT1X_HUMAN	Metallothionein-1X	61	0	3	1.592
421	TXD17_HUMAN	Thioredoxin domain-containing protein 17	123	0	3	1.592
422	TCPW_HUMAN	T-complex protein 1 subunit zeta-2	530	0	3	1.592
423	ANX10_HUMAN	Annexin A10	324	0	3	1.592
424	CATD_HUMAN	Cathepsin D	412	0	3	1.592
425	AK1BA_HUMAN	Aldo-keto reductase family 1 member B10	316	0	3	1.592
426	SMD3_HUMAN	Small nuclear ribonucleoprotein Sm D3	126	0	3	1.592
427	NONO_HUMAN	Non-POU domain-containing octamer-binding protein	471	0	3	1.592
428	PHB_HUMAN	Prohibitin	272	0	3	1.592
429	ZN185_HUMAN	Zinc finger protein 185	689	0	3	1.592
430	XRCC5_HUMAN	X-ray repair cross-complementing protein 5	732	0	3	1.592

431	TPD54_HUMAN	Tumor protein D54	206	0	3	1.592
432	S10AE_HUMAN	Protein S100-A14	104	0	3	1.592
433	DESP_HUMAN	Desmoplakin	2871	0	3	1.592
434	DHRS9_HUMAN	Dehydrogenase/reductase SDR family member 9	319	0	3	1.592
435	STIP1_HUMAN	Stress-induced-phosphoprotein 1	543	0	3	1.592
436	ZN609_HUMAN	Zinc finger protein 609	1411	0	3	1.592
437	K2C5_HUMAN	Keratin, type II cytoskeletal 5	590	25	95	1.714
438	K1C15_HUMAN	Keratin, type I cytoskeletal 15	456	11	44	1.717
439	HSP71_HUMAN	Heat shock 70 kDa protein 1A/1B	641	7	30	1.751
440	CNN2_HUMAN	Calponin-2	309	1	8	1.866
441	KT222_HUMAN	Keratin-like protein KRT222	295	0	4	1.897
442	K1H2_HUMAN	Keratin, type I cuticular Ha2	448	0	4	1.897
443	H2AX_HUMAN	Histone H2AX	143	0	4	1.897
444	RA1L2_HUMAN	Heterogeneous nuclear ribonucleoprotein A1-like 2	320	0	4	1.897
445	ADT4_HUMAN	ADP/ATP translocase 4	315	0	4	1.897
446	RS26_HUMAN	40S ribosomal protein S26	115	0	4	1.897
447	CRIP1_HUMAN	Cysteine-rich protein 1	77	0	4	1.897
448	CAN1_HUMAN	Calpain-1 catalytic subunit	714	0	4	1.897
449	CTNA1_HUMAN	Catenin alpha-1	906	0	4	1.897
450	SET_HUMAN	Protein SET	290	0	4	1.897
451	LIMA1_HUMAN	LIM domain and actin-binding protein 1	759	0	4	1.897
452	RAB7A_HUMAN	Ras-related protein Rab-7a	207	0	4	1.897
453	K2C8_HUMAN	Keratin, type II cytoskeletal 8	483	20	92	1.973
454	KRT35_HUMAN	Keratin, type I cuticular Ha5	455	0	5	2.148
455	CBX3_HUMAN	Chromobox protein homolog 3	183	0	5	2.148
456	AL1A3_HUMAN	Aldehyde dehydrogenase family 1 member A3	512	0	5	2.148
457	PDIA4_HUMAN	Protein disulfide-isomerase A4	645	0	5	2.148
458	AGR2_HUMAN	Anterior gradient protein 2 homolog	175	0	5	2.148
459	AATM_HUMAN	Aspartate aminotransferase, mitochondrial	430	0	5	2.148

460	PNPH_HUMAN	Purine nucleoside phosphorylase	289	0	5	2.148
461	K2C7_HUMAN	Keratin, type II cytoskeletal 7	469	16	92	2.275
462	SPB5_HUMAN	Serpin B5	375	0	6	2.363
463	G6PI_HUMAN	Glucose-6-phosphate isomerase	558	2	18	2.395
464	K1H1_HUMAN	Keratin, type I cuticular Ha1	416	0	7	2.549
465	ENOG_HUMAN	Gamma-enolase	434	0	7	2.549
466	GDIR2_HUMAN	Rho GDP-dissociation inhibitor 2	201	0	7	2.549
467	H2B1M_HUMAN	Histone H2B type 1-M	126	0	7	2.549
468	NUCL_HUMAN	Nucleolin	710	1	14	2.589
469	H2B1O_HUMAN	Histone H2B type 1-O	126	0	8	2.715
470	S10A2_HUMAN	Protein S100-A2	98	0	8	2.715
471	K1C23_HUMAN	Keratin, type I cytoskeletal 23	422	0	9	2.863
472	PCNA_HUMAN	Proliferating cell nuclear antigen	261	0	11	3.120
473	K1C20_HUMAN	Keratin, type I cytoskeletal 20	424	0	12	3.234
474	G6PD_HUMAN	Glucose-6-phosphate 1-dehydrogenase	515	0	12	3.234
475	RLA0_HUMAN	60S acidic ribosomal protein P0	317	0	12	3.234
476	K1C18_HUMAN	Keratin, type I cytoskeletal 18	430	3	46	3.309
477	FSCN1_HUMAN	Fascin	493	1	25	3.375
478	K1C17_HUMAN	Keratin, type I cytoskeletal 17	432	7	104	3.519
479	K2C72_HUMAN	Keratin, type II cytoskeletal 72	511	0	19	3.847
480	PLSL_HUMAN	Plastin-2	627	0	19	3.847
481	RADI_HUMAN	Radixin	583	0	21	3.984
482	K1C19_HUMAN	Keratin, type I cytoskeletal 19	400	4	113	4.292
483	1433S_HUMAN	14-3-3 protein sigma	248	0	33	4.608
484	ACTG_HUMAN	Actin, cytoplasmic 2	375	0	41	4.913

Expression levels of these 484 proteins were more than 2-fold higher or lower in BxPC-3 cells compared to HPNE cells.

Table S2. Differentially expressed proteins in PANC-1 cells.

No.	ID	Protein name	Number of amino acids	Spectral counting		
				HPNE	PANC-1	Fold chang (Rsc)
1	FINC_HUMAN	Fibronectin	2386	109	0	-6.532
2	TAGL_HUMAN	Transgelin	201	38	0	-5.025
3	K1C16_HUMAN	Keratin, type I cytoskeletal 16	473	26	0	-4.496
4	PAI1_HUMAN	Plasminogen activator inhibitor 1	402	20	0	-4.136
5	H2B1H_HUMAN	Histone H2B type 1-H	126	18	0	-3.992
6	ANXA6_HUMAN	Annexin A6	673	16	0	-3.834
7	EF1A3_HUMAN	Putative elongation factor 1-alpha-like 3	462	15	0	-3.747
8	LASP1_HUMAN	LIM and SH3 domain protein 1	261	14	0	-3.655
9	CALD1_HUMAN	Caldesmon	793	14	0	-3.655
10	ARF4_HUMAN	ADP-ribosylation factor 4	180	14	0	-3.655
11	CKAP4_HUMAN	Cytoskeleton-associated protein 4	602	10	0	-3.216
12	CO1A1_HUMAN	Collagen alpha-1(I) chain	1464	10	0	-3.216
13	TPM4_HUMAN	Tropomyosin alpha-4 chain	248	36	3	-3.183
14	MYH10_HUMAN	Myosin-10	1976	18	1	-3.144
15	H2A2C_HUMAN	Histone H2A type 2-C	129	8	0	-2.933
16	RAB1B_HUMAN	Ras-related protein Rab-1B	201	8	0	-2.933
17	WDR1_HUMAN	WD repeat-containing protein 1	606	8	0	-2.933
18	H2B1C_HUMAN	Histone H2B type 1-C/E/F/G/I	126	8	0	-2.933
19	TPM2_HUMAN	Tropomyosin beta chain	284	29	3	-2.881
20	ANXA1_HUMAN	Annexin A1	346	34	4	-2.798
21	FLNC_HUMAN	Filamin-C	2725	27	3	-2.782
22	GDIR1_HUMAN	Rho GDP-dissociation inhibitor 1	204	7	0	-2.768
23	H2B1J_HUMAN	Histone H2B type 1-J	126	7	0	-2.768
24	TGM2_HUMAN	Protein-glutamine gamma-glutamyltransferase 2	687	6	0	-2.581

25	RAB10_HUMAN	Ras-related protein Rab-10	200	6	0	-2.581
26	MT1G_HUMAN	Metallothionein-1G	62	6	0	-2.581
27	CBR1_HUMAN	Carbonyl reductase [NADPH] 1	277	6	0	-2.581
28	MVP_HUMAN	Major vault protein	893	6	0	-2.581
29	MT2_HUMAN	Metallothionein-2	61	6	0	-2.581
30	FLNA_HUMAN	Filamin-A	2647	88	16	-2.432
31	ARF3_HUMAN	ADP-ribosylation factor 3	181	5	0	-2.367
32	TPM1_HUMAN	Tropomyosin alpha-1 chain	284	25	5	-2.118
33	PLST_HUMAN	Plastin-3	630	4	0	-2.115
34	HSPB6_HUMAN	Heat shock protein beta-6	160	4	0	-2.115
35	CALU_HUMAN	Calumenin	315	4	0	-2.115
36	GNAI2_HUMAN	Guanine nucleotide-binding protein G(i) subunit alpha-2	355	4	0	-2.115
37	RL35_HUMAN	60S ribosomal protein L35	123	4	0	-2.115
38	MYH11_HUMAN	Myosin-11 PE=1 SV=3	1972	22	5	-1.943
39	SERPH_HUMAN	Serpin H1	418	22	5	-1.943
40	MYH14_HUMAN	Myosin-14	1995	21	5	-1.879
41	MYH13_HUMAN	Myosin-13	1938	3	0	-1.810
42	CLH2_HUMAN	Clathrin heavy chain 2	1640	3	0	-1.810
43	RAB12_HUMAN	Ras-related protein Rab-12	244	3	0	-1.810
44	CO6A3_HUMAN	Collagen alpha-3(VI) chain	3177	3	0	-1.810
45	CPNS1_HUMAN	Calpain small subunit 1	268	3	0	-1.810
46	CBR3_HUMAN	Carbonyl reductase [NADPH] 3	277	3	0	-1.810
47	CAV1_HUMAN	Caveolin-1	178	3	0	-1.810
48	TMOD2_HUMAN	Tropomodulin-2	351	3	0	-1.810
49	UCHL1_HUMAN	Ubiquitin carboxyl-terminal hydrolase isozyme L1	223	3	0	-1.810
50	SPI2_HUMAN	Serpin I2	405	3	0	-1.810
51	TRI10_HUMAN	Tripartite motif-containing protein 10	481	3	0	-1.810
52	DYHC1_HUMAN	Cytoplasmic dynein 1 heavy chain 1	4646	3	0	-1.810
53	FBLN1_HUMAN	Fibulin-1	703	3	0	-1.810

54	IF5A2_HUMAN	Eukaryotic translation initiation factor 5A-2	153	3	0	-1.810
55	MYH9_HUMAN	Myosin-9	1960	125	37	-1.787
56	GELS_HUMAN	Gelsolin	782	9	2	-1.702
57	DCD_HUMAN	Dermcidin	110	12	3	-1.686
58	CALR_HUMAN	Calreticulin	417	14	4	-1.584
59	PLEC_HUMAN	Plectin	4684	28	9	-1.561
60	PGAM2_HUMAN	Phosphoglycerate mutase 2	253	8	2	-1.554
61	ENPLL_HUMAN	Putative endoplasmin-like protein	399	5	1	-1.518
62	CAN2_HUMAN	Calpain-2 catalytic subunit	700	5	1	-1.518
63	CLH1_HUMAN	Clathrin heavy chain 1	1675	13	4	-1.486
64	K2C74_HUMAN	Keratin, type II cytoskeletal 74	529	10	3	-1.449
65	GRHL1_HUMAN	Grainyhead-like protein 1 homolog	618	2	0	-1.422
66	ARPC5_HUMAN	Actin-related protein 2/3 complex subunit 5	151	2	0	-1.422
67	TPT1L_HUMAN	TPT1-like protein	140	2	0	-1.422
68	CSRP1_HUMAN	Cysteine and glycine-rich protein 1	193	2	0	-1.422
69	RRBP1_HUMAN	Ribosome-binding protein 1	1410	2	0	-1.422
70	MTAP2_HUMAN	Microtubule-associated protein 2	1827	2	0	-1.422
71	ECHB_HUMAN	Trifunctional enzyme subunit beta, mitochondrial	474	2	0	-1.422
72	SMC5_HUMAN	Structural maintenance of chromosomes protein 5	1101	2	0	-1.422
73	MAP1B_HUMAN	Microtubule-associated protein 1B	2468	2	0	-1.422
74	CCD33_HUMAN	Coiled-coil domain-containing protein 33	958	2	0	-1.422
75	PLSI_HUMAN	Plastin-1	629	2	0	-1.422
76	SARG_HUMAN	Specifically androgen-regulated gene protein	601	2	0	-1.422
77	NNMT_HUMAN	Nicotinamide N-methyltransferase	264	2	0	-1.422
78	RL14_HUMAN	60S ribosomal protein L14	215	2	0	-1.422
79	KRT82_HUMAN	Keratin, type II cuticular Hb2	513	2	0	-1.422
80	MYL9_HUMAN	Myosin regulatory light polypeptide 9	172	2	0	-1.422
81	CHD4_HUMAN	Chromodomain-helicase-DNA-binding protein 4	1912	2	0	-1.422
82	XPP3_HUMAN	Probable Xaa-Pro aminopeptidase 3	507	2	0	-1.422

83	KATL2_HUMAN	Katanin p60 ATPase-containing subunit A-like 2	538	2	0	-1.422
84	GNAI1_HUMAN	Guanine nucleotide-binding protein G(i) subunit alpha-1	354	2	0	-1.422
85	HORN_HUMAN	Hornerin	2850	2	0	-1.422
86	HXK1_HUMAN	Hexokinase-1	917	2	0	-1.422
87	FKB10_HUMAN	Peptidyl-prolyl cis-trans isomerase FKBP10	582	2	0	-1.422
88	CAZA2_HUMAN	F-actin-capping protein subunit alpha-2	286	2	0	-1.422
89	SPTB2_HUMAN	Spectrin beta chain, non-erythrocytic 1	2364	2	0	-1.422
90	MDR1_HUMAN	Multidrug resistance protein 1	1280	2	0	-1.422
91	BCL7B_HUMAN	B-cell CLL/lymphoma 7 protein family member B	202	2	0	-1.422
92	CO3A1_HUMAN	Collagen alpha-1(III) chain	1466	2	0	-1.422
93	ARPC2_HUMAN	Actin-related protein 2/3 complex subunit 2	300	2	0	-1.422
94	RS15_HUMAN	40S ribosomal protein S15	145	2	0	-1.422
95	IDHC_HUMAN	Isocitrate dehydrogenase [NADP] cytoplasmic	414	2	0	-1.422
96	SYCP2_HUMAN	Synaptonemal complex protein 2	1530	2	0	-1.422
97	VIGLN_HUMAN	Vigilin	1268	2	0	-1.422
98	NSF1C_HUMAN	NSFL1 cofactor p47	370	2	0	-1.422
99	CNTRL_HUMAN	Centriolin	2325	2	0	-1.422
100	RAB1A_HUMAN	Ras-related protein Rab-1A	205	7	2	-1.389
101	RHOA_HUMAN	Transforming protein RhoA	193	7	2	-1.389
102	PRDX6_HUMAN	Peroxiredoxin-6	224	12	4	-1.381
103	TPM3_HUMAN	Tropomyosin alpha-3 chain	285	24	9	-1.348
104	HBA_HUMAN	Hemoglobin subunit alpha	142	9	3	-1.315
105	MT1E_HUMAN	Metallothionein-1E	61	4	1	-1.267
106	RL10A_HUMAN	60S ribosomal protein L10a	217	4	1	-1.267
107	RS8_HUMAN	40S ribosomal protein S8	208	4	1	-1.267
108	RL19_HUMAN	60S ribosomal protein L19	196	4	1	-1.267
109	RPN1_HUMAN	Dolichyl-diphosphooligosaccharide--protein glycosyltransferase subunit 1	607	4	1	-1.267
110	SH3L3_HUMAN	SH3 domain-binding glutamic acid-rich-like protein 3	93	6	2	-1.202
111	K1C26_HUMAN	Keratin, type I cytoskeletal 26	468	9	4	-1.010

112	RLA0L_HUMAN	60S acidic ribosomal protein P0-like	317	9	4	-1.010
113	RL8_HUMAN	60S ribosomal protein L8	257	7	3	-1.001
114	RS11_HUMAN	40S ribosomal protein S11	158	7	3	-1.001
115	H2A1B_HUMAN	Histone H2A type 1-B/E	130	9	20	1.011
116	RAN_HUMAN	GTP-binding nuclear protein Ran	216	7	16	1.023
117	ENOA_HUMAN	Alpha-enolase	434	29	63	1.051
118	ENOB_HUMAN	Beta-enolase	434	4	10	1.057
119	VINC_HUMAN	Vinculin	1134	9	21	1.077
120	K1C17_HUMAN	Keratin, type I cytoskeletal	432	7	17	1.104
121	SPTN4_HUMAN	Spectrin beta chain, non-erythrocytic 4	2564	2	6	1.115
122	NACAM_HUMAN	Nascent polypeptide-associated complex subunit alpha, muscle-specific form	2078	2	6	1.115
123	PRDX3_HUMAN	Thioredoxin-dependent peroxide reductase, mitochondrial	256	2	6	1.115
124	PTMA_HUMAN	Prothymosin alpha	111	2	6	1.115
125	ROA1_HUMAN	Heterogeneous nuclear ribonucleoprotein A1	372	2	6	1.115
126	HMGB1_HUMAN	High mobility group protein B1	215	2	6	1.115
127	ACTN4_HUMAN	Alpha-actinin-4	911	48	108	1.119
128	EF1D_HUMAN	Elongation factor 1-delta	281	6	15	1.123
129	HNRPF_HUMAN	Heterogeneous nuclear ribonucleoprotein F	415	1	4	1.180
130	S10A6_HUMAN	Protein S100-A6	90	1	4	1.180
131	PA2G4_HUMAN	Proliferation-associated protein 2G4	394	1	4	1.180
132	H90B4_HUMAN	Putative heat shock protein HSP 90-beta 4	505	1	4	1.180
133	ROA2_HUMAN	Heterogeneous nuclear ribonucleoproteins A2/B1	353	9	23	1.202
134	RL12_HUMAN	60S ribosomal protein L12	165	3	9	1.228
135	PGK1_HUMAN	Phosphoglycerate kinase 1	417	14	36	1.250
136	TBA4B_HUMAN	Putative tubulin-like protein alpha-4B	241	0	2	1.336
137	K1H1_HUMAN	Keratin, type I cuticular Ha1	416	0	2	1.336
138	LMNB2_HUMAN	Lamin-B2	600	0	2	1.336
139	DX39B_HUMAN	Spliceosome RNA helicase DDX39B	428	0	2	1.336
140	IF2B1_HUMAN	Insulin-like growth factor 2 mRNA-binding protein	577	0	2	1.336

141	HNRPR_HUMAN	Heterogeneous nuclear ribonucleoprotein R	633	0	2	1.336
142	DDX5_HUMAN	Probable ATP-dependent RNA helicase DDX5	614	0	2	1.336
143	G3BP1_HUMAN	Ras GTPase-activating protein-binding protein 1	466	0	2	1.336
144	CAPG_HUMAN	Macrophage-capping protein	348	0	2	1.336
145	SMD3_HUMAN	Small nuclear ribonucleoprotein Sm D3	126	0	2	1.336
146	CAZA1_HUMAN	F-actin-capping protein subunit alpha-1	286	0	2	1.336
147	S10A4_HUMAN	Protein S100-A4	101	0	2	1.336
148	PUR2_HUMAN	Trifunctional purine biosynthetic protein adenosine-3	1010	0	2	1.336
149	TRY2_HUMAN	Trypsin-2	247	0	2	1.336
150	SRSF4_HUMAN	Serine/arginine-rich splicing factor 4	494	0	2	1.336
151	AATM_HUMAN	Aspartate aminotransferase, mitochondrial	430	0	2	1.336
152	CYB5B_HUMAN	Cytochrome b5 type B	146	0	2	1.336
153	RPN2_HUMAN	Dolichyl-diphosphooligosaccharide--protein glycosyltransferase subunit 2	631	0	2	1.336
154	EIF3A_HUMAN	Eukaryotic translation initiation factor 3 subunit A	1382	0	2	1.336
155	RS5_HUMAN	40S ribosomal protein S5	204	0	2	1.336
156	EIF3H_HUMAN	Eukaryotic translation initiation factor 3 subunit H	352	0	2	1.336
157	GRHPR_HUMAN	Glyoxylate reductase/hydroxypyruvate reductase	328	0	2	1.336
158	LGUL_HUMAN	Lactoylglutathione lyase	184	0	2	1.336
159	IF6_HUMAN	Eukaryotic translation initiation factor 6	245	0	2	1.336
160	RS28_HUMAN	40S ribosomal protein S28	69	0	2	1.336
161	RAB7A_HUMAN	Ras-related protein Rab-7a	207	0	2	1.336
162	ZN397_HUMAN	Zinc finger protein 397	534	0	2	1.336
163	AKT2_HUMAN	RAC-beta serine/threonine-protein kinase	481	0	2	1.336
164	CTL3_HUMAN	Choline transporter-like protein 3	653	0	2	1.336
165	RL29_HUMAN	60S ribosomal protein L29	159	0	2	1.336
166	SYNC_HUMAN	Asparagine--tRNA ligase, cytoplasmic	548	0	2	1.336
167	MT1X_HUMAN	Metallothionein-1X	61	0	2	1.336
168	ATPO_HUMAN	ATP synthase subunit O, mitochondrial	213	0	2	1.336
169	PYGL_HUMAN	Glycogen phosphorylase, liver form	847	0	2	1.336

170	SPSY_HUMAN	Spermine synthase	366	0	2	1.336
171	TXD17_HUMAN	Thioredoxin domain-containing protein 17	123	0	2	1.336
172	SP100_HUMAN	Nuclear autoantigen Sp-100	879	0	2	1.336
173	XRCC6_HUMAN	X-ray repair cross-complementing protein 6	609	0	2	1.336
174	HPCL1_HUMAN	Hippocalcin-like protein 1	193	0	2	1.336
175	LAMP2_HUMAN	Lysosome-associated membrane glycoprotein 2	410	0	2	1.336
176	CC180_HUMAN	Coiled-coil domain-containing protein 180	1646	0	2	1.336
177	MCM2_HUMAN	DNA replication licensing factor MCM2	904	0	2	1.336
178	COTL1_HUMAN	Coactosin-like protein	142	0	2	1.336
179	TRIM1_HUMAN	Probable E3 ubiquitin-protein ligase MID2	735	0	2	1.336
180	HNRH3_HUMAN	Heterogeneous nuclear ribonucleoprotein H3	346	0	2	1.336
181	AT2B3_HUMAN	Plasma membrane calcium-transporting ATPase 3	1220	0	2	1.336
182	CHM4B_HUMAN	Charged multivesicular body protein 4b	224	0	2	1.336
183	HS74L_HUMAN	Heat shock 70 kDa protein 4L	839	0	2	1.336
184	HYOU1_HUMAN	Hypoxia up-regulated protein 1	999	0	2	1.336
185	TAXB1_HUMAN	Tax1-binding protein 1	789	0	2	1.336
186	CAN1_HUMAN	Calpain-1 catalytic subunit	714	0	2	1.336
187	HNRC1_HUMAN	Heterogeneous nuclear ribonucleoprotein C-like 1	293	3	10	1.363
188	NPM_HUMAN	Nucleophosmin	294	9	26	1.371
189	RINI_HUMAN	Ribonuclease inhibitor	461	1	5	1.431
190	PYGB_HUMAN	Glycogen phosphorylase, brain form	843	1	5	1.431
191	CH10_HUMAN	10 kDa heat shock protein, mitochondrial	102	6	19	1.441
192	PARK7_HUMAN	Protein DJ-1	189	3	11	1.486
193	TCPD_HUMAN	T-complex protein 1 subunit delta	539	4	14	1.497
194	HNRPC_HUMAN	Heterogeneous nuclear ribonucleoproteins C1/C2	306	7	23	1.516
195	TCPG_HUMAN	T-complex protein 1 subunit gamma	545	5	18	1.582
196	TCPZ_HUMAN	T-complex protein 1 subunit zeta	531	4	15	1.589
197	G6PI_HUMAN	Glucose-6-phosphate isomerase	558	2	9	1.615
198	H2A2B_HUMAN	Histone H2A type 2-B	130	2	9	1.615

199	TCPE_HUMAN	T-complex protein 1 subunit epsilon	541	2	9	1.615
200	IPO7_HUMAN	Importin-7	1038	2	9	1.615
201	FSCN1_HUMAN	Fascin	493	1	6	1.646
202	GLU2B_HUMAN	Glucosidase 2 subunit beta	528	1	6	1.646
203	1433S_HUMAN	14-3-3 protein	248	0	3	1.723
204	NP1L4_HUMAN	Nucleosome assembly protein 1-like 4	375	0	3	1.723
205	SERC_HUMAN	Phosphoserine aminotransferase	370	0	3	1.723
206	PFD2_HUMAN	Prefoldin subunit 2	154	0	3	1.723
207	PA1B2_HUMAN	Platelet-activating factor acetylhydrolase IB subunit beta	229	0	3	1.723
208	RL10_HUMAN	60S ribosomal protein L10	214	0	3	1.723
209	SQRD_HUMAN	Sulfide:quinone oxidoreductase, mitochondrial	450	0	3	1.723
210	SRSF2_HUMAN	Serine/arginine-rich splicing factor 2	221	0	3	1.723
211	SRSF6_HUMAN	Serine/arginine-rich splicing factor 6	344	0	3	1.723
212	CRIP1_HUMAN	Cysteine-rich protein 1	77	0	3	1.723
213	PTBP1_HUMAN	Polypyrimidine tract-binding protein 1	531	0	3	1.723
214	HN1_HUMAN	Hematological and neurological expressed 1 protein	154	0	3	1.723
215	O2T35_HUMAN	Olfactory receptor 2T35	323	0	3	1.723
216	LAP2A_HUMAN	Lamina-associated polypeptide 2, isoform alpha	694	0	3	1.723
217	RBM3_HUMAN	Putative RNA-binding protein 3	157	0	3	1.723
218	RUVB2_HUMAN	RuvB-like 2	463	0	3	1.723
219	RUXE_HUMAN	Small nuclear ribonucleoprotein E	92	0	3	1.723
220	STIP1_HUMAN	Stress-induced-phosphoprotein 1	543	0	3	1.723
221	SMD1_HUMAN	Small nuclear ribonucleoprotein Sm D1	119	0	3	1.723
222	DIAP3_HUMAN	Protein diaphanous homolog 3	1193	0	3	1.723
223	ST134_HUMAN	Putative protein FAM10A4	240	0	3	1.723
224	NACP1_HUMAN	Putative nascent polypeptide-associated complex subunit alpha-like protein	213	0	3	1.723
225	PHB_HUMAN	Prohibitin	272	0	3	1.723
226	FKB1A_HUMAN	Peptidyl-prolyl cis-trans isomerase FKBP1A	108	0	3	1.723
227	TM9S3_HUMAN	Transmembrane 9 superfamily member 3	589	0	3	1.723

228	PDIA4_HUMAN	Protein disulfide-isomerase A4	645	0	3	1.723
229	CH60_HUMAN	60 kDa heat shock protein, mitochondrial	573	15	56	1.783
230	TCPQ_HUMAN	T-complex protein 1 subunit theta	548	3	14	1.802
231	RL22_HUMAN	60S ribosomal protein L22	128	1	7	1.832
232	TCPA_HUMAN	T-complex protein 1 subunit alpha	556	4	19	1.907
233	ENOG_HUMAN	Gamma-enolase	434	0	4	2.028
234	TCPW_HUMAN	T-complex protein 1 subunit zeta-2	530	0	4	2.028
235	RA1L2_HUMAN	Heterogeneous nuclear ribonucleoprotein A1-like 2	320	0	4	2.028
236	F10A1_HUMAN	Hsc70-interacting protein	369	0	4	2.028
237	HPRT_HUMAN	Hypoxanthine-guanine phosphoribosyltransferase	218	0	4	2.028
238	EFTU_HUMAN	Elongation factor Tu, mitochondrial	452	0	4	2.028
239	ADT4_HUMAN	ADP/ATP translocase 4	315	0	4	2.028
240	G6PD_HUMAN	Glucose-6-phosphate 1-dehydrogenase	515	0	4	2.028
241	RAP1B_HUMAN	Ras-related protein Rap-1b	184	0	4	2.028
242	NONO_HUMAN	Non-POU domain-containing octamer-binding protein	471	0	4	2.028
243	AHNK2_HUMAN	Protein AHNAK2	5795	0	4	2.028
244	K2C72_HUMAN	Keratin, type II cytoskeletal 72	511	0	4	2.028
245	KRT35_HUMAN	Keratin, type I cuticular Ha5	455	0	4	2.028
246	SRC8_HUMAN	Src substrate cortactin	550	0	4	2.028
247	FBRL_HUMAN	rRNA 2~-O-methyltransferase fibrillarin	321	0	4	2.028
248	EST1_HUMAN	Liver carboxylesterase 1	567	0	4	2.028
249	TCPH_HUMAN	T-complex protein 1 subunit eta	543	2	13	2.091
250	K2C80_HUMAN	Keratin, type II cytoskeletal 80	452	1	9	2.146
251	TBCA_HUMAN	Tubulin-specific chaperone A	108	1	9	2.146
252	K2C8_HUMAN	Keratin, type II cytoskeletal 8	483	20	98	2.198
253	GRP75_HUMAN	Stress-70 protein, mitochondrial	679	4	26	2.337
254	RLA0_HUMAN	60S acidic ribosomal protein P0	317	0	6	2.494
255	HNRPL_HUMAN	Heterogeneous nuclear ribonucleoprotein L	589	0	6	2.494
256	TCPB_HUMAN	T-complex protein 1 subunit beta	535	2	19	2.600

257	H2B1D_HUMAN	Histone H2B type 1-D	126	0	7	2.681
258	LPPRC_HUMAN	Leucine-rich PPR motif-containing protein, mitochondrial	1394	0	7	2.681
259	H2B1M_HUMAN	Histone H2B type 1-M	126	0	7	2.681
260	CATD_HUMAN	Cathepsin D	412	0	8	2.846
261	XRCC5_HUMAN	X-ray repair cross-complementing protein 5	732	0	8	2.846
262	MDHM_HUMAN	Malate dehydrogenase, mitochondrial	338	2	25	2.976
263	H2AX_HUMAN	Histone H2AX	143	0	9	2.994
264	ECH1_HUMAN	Delta(3,5)-Delta(2,4)-dienoyl-CoA isomerase, mitochondrial	328	0	9	2.994
265	H2B1K_HUMAN	Histone H2B type 1-K	126	0	9	2.994
266	H2A1J_HUMAN	Histone H2A type 1-J	128	0	10	3.129
267	NP1L1_HUMAN	Nucleosome assembly protein 1-like 1	391	0	10	3.129
268	TIM50_HUMAN	Mitochondrial import inner membrane translocase subunit TIM50	353	0	10	3.129
269	K1C18_HUMAN	Keratin, type I cytoskeletal 18	430	3	40	3.244
270	NUCL_HUMAN	Nucleolin OS=Homo sapiens	710	1	21	3.267
271	K1C19_HUMAN	Keratin, type I cytoskeletal 19	400	4	54	3.363
272	RADI_HUMAN	Radixin	583	0	15	3.660
273	ACTG_HUMAN	Actin, cytoplasmic 2	375	0	110	6.457

Expression levels of these 273 proteins were more than 2-fold higher or lower in PANC-1 cells compared to HPNE cells.

Table S3. Differentially expressed proteins in pancreatic cancer cells.

No.	ID	Protein name	fold change (Rsc)	
			BxpC-3	PANC-1
1	FINC_HUMAN	Fibronectin	-5.814	-6.532
2	TAGL_HUMAN	Transgelin	-5.156	-5.025
3	TPM2_HUMAN	Tropomyosin beta chain	-4.778	-2.881
4	PAI1_HUMAN	Plasminogen activator inhibitor 1	-4.267	-4.136
5	ANXA6_HUMAN	Annexin A6	-3.965	-3.834
6	EF1A3_HUMAN	Putative elongation factor 1-alpha-like 3	-3.878	-3.747
7	CALD1_HUMAN	Caldesmon	-3.787	-3.655
8	CKAP4_HUMAN	Cytoskeleton-associated protein 4	-3.347	-3.216
9	CO1A1_HUMAN	Collagen alpha-1(I) chain	-3.347	-3.216
10	RAB1B_HUMAN	Ras-related protein Rab-1B	-3.064	-2.933
11	PGAM2_HUMAN	Phosphoglycerate mutase 2	-3.064	-1.554
12	TPM4_HUMAN	Tropomyosin alpha-4 chain	-3.009	-3.183
13	H2B1J_HUMAN	Histone H2B type 1-J	-2.899	-2.768
14	RAB1A_HUMAN	Ras-related protein Rab-1A	-2.899	-1.389
15	MT1G_HUMAN	Metallothionein-1G	-2.712	-2.581
16	CBR1_HUMAN	Carbonyl reductase [NADPH] 1	-2.712	-2.581
17	MT2_HUMAN	Metallothionein-2	-2.712	-2.581
18	ARF3_HUMAN	ADP-ribosylation factor 3	-2.498	-2.367
19	HSPB6_HUMAN	Heat shock protein beta-6	-2.246	-2.115
20	CALU_HUMAN	Calumenin	-2.246	-2.115
21	GNAI2_HUMAN	Guanine nucleotide-binding protein G(i) subunit alpha-2	-2.246	-2.115
22	RL35_HUMAN	60S ribosomal protein L35	-2.246	-2.115
23	RL19_HUMAN	60S ribosomal protein L19	-2.246	-1.267
24	WDR1_HUMAN	WD repeat-containing protein 1	-2.216	-2.933
25	GDIR1_HUMAN	Rho GDP-dissociation inhibitor 1	-2.050	-2.768

26	CO6A3_HUMAN	Collagen alpha-3(VI) chain	-1.941	-1.810
27	CBR3_HUMAN	Carbonyl reductase [NADPH] 3	-1.941	-1.810
28	UCHL1_HUMAN	Ubiquitin carboxyl-terminal hydrolase isozyme L1	-1.941	-1.810
29	SPI2_HUMAN	Serpin I2	-1.941	-1.810
30	TRI10_HUMAN	Tripartite motif-containing protein 10	-1.941	-1.810
31	FBLN1_HUMAN	Fibulin-1	-1.941	-1.810
32	MVP_HUMAN	Major vault protein	-1.864	-2.581
33	TPM1_HUMAN	Tropomyosin alpha-1 chain	-1.849	-2.118
34	GELS_HUMAN	Gelsolin	-1.833	-1.702
35	PRDX6_HUMAN	Peroxiredoxin-6	-1.817	-1.381
36	LASP1_HUMAN	LIM and SH3 domain protein 1	-1.715	-3.655
37	CAN2_HUMAN	Calpain-2 catalytic subunit	-1.649	-1.518
38	MYH14_HUMAN	Myosin-14	-1.609	-1.879
39	GRHL1_HUMAN	Grainyhead-like protein 1 homolog	-1.554	-1.422
40	ARPC5_HUMAN	Actin-related protein 2/3 complex subunit 5	-1.554	-1.422
41	CSRP1_HUMAN	Cysteine and glycine-rich protein 1	-1.554	-1.422
42	RRBP1_HUMAN	Ribosome-binding protein 1	-1.554	-1.422
43	MTAP2_HUMAN	Microtubule-associated protein 2	-1.554	-1.422
44	ECHB_HUMAN	Trifunctional enzyme subunit beta, mitochondrial	-1.554	-1.422
45	SMC5_HUMAN	Structural maintenance of chromosomes protein 5	-1.554	-1.422
46	MAP1B_HUMAN	Microtubule-associated protein 1B	-1.554	-1.422
47	CCD33_HUMAN	Coiled-coil domain-containing protein 33	-1.554	-1.422
48	NNMT_HUMAN	Nicotinamide N-methyltransferase	-1.554	-1.422
49	KRT82_HUMAN	Keratin, type II cuticular Hb2	-1.554	-1.422
50	CHD4_HUMAN	Chromodomain-helicase-DNA-binding protein 4	-1.554	-1.422
51	XPP3_HUMAN	Probable Xaa-Pro aminopeptidase 3	-1.554	-1.422
52	KATL2_HUMAN	Katanin p60 ATPase-containing subunit A-like 2	-1.554	-1.422
53	GNAI1_HUMAN	Guanine nucleotide-binding protein G(i) subunit alpha-1	-1.554	-1.422
54	FKB10_HUMAN	Peptidyl-prolyl cis-trans isomerase FKBP10	-1.554	-1.422

55	BCL7B_HUMAN	B-cell CLL/lymphoma 7 protein family member B	-1.554	-1.422
56	CO3A1_HUMAN	Collagen alpha-1(III) chain	-1.554	-1.422
57	RS15_HUMAN	40S ribosomal protein S15	-1.554	-1.422
58	IDHC_HUMAN	Isocitrate dehydrogenase [NADP] cytoplasmic	-1.554	-1.422
59	SYCP2_HUMAN	Synaptonemal complex protein 2	-1.554	-1.422
60	VIGLN_HUMAN	Vigilin	-1.554	-1.422
61	NSF1C_HUMAN	NSFL1 cofactor p47	-1.554	-1.422
62	CNTRL_HUMAN	Centriolin	-1.554	-1.422
63	IPO7_HUMAN	Importin-7	-1.554	1.615
64	DCD_HUMAN	Dermcidin	-1.512	-1.686
65	MYH10_HUMAN	Myosin-10	-1.400	-3.144
66	MT1E_HUMAN	Metallothionein-1E	-1.398	-1.267
67	RS8_HUMAN	40S ribosomal protein S8	-1.398	-1.267
68	CLH1_HUMAN	Clathrin heavy chain 1	-1.366	-1.486
69	RAB10_HUMAN	Ras-related protein Rab-10	-1.333	-2.581
70	CALR_HUMAN	Calreticulin	-1.249	-1.584
71	MYH11_HUMAN	Myosin-11	-1.225	-1.943
72	RL8_HUMAN	60S ribosomal protein L8	-1.133	-1.001
73	CLH2_HUMAN	Clathrin heavy chain 2	-1.093	-1.810
74	RAB12_HUMAN	Ras-related protein Rab-12	-1.093	-1.810
75	H2B1H_HUMAN	Histone H2B type 1-H	-1.086	-3.992
76	MYH9_HUMAN	Myosin-9	-1.077	-1.787
77	FLNC_HUMAN	Filamin-C	-1.067	-2.782
78	ARF4_HUMAN	ADP-ribosylation factor 4	-1.063	-3.655
79	RINI_HUMAN	Ribonuclease inhibitor	-1.023	1.431
80	PYGB_HUMAN	Glycogen phosphorylase, brain form	-1.023	1.431
81	TBCA_HUMAN	Tubulin-specific chaperone A	-1.023	2.146
82	HNRPF_HUMAN	Heterogeneous nuclear ribonucleoprotein F	1.048	1.180
83	PA2G4_HUMAN	Proliferation-associated protein 2G4	1.048	1.180

84	RL22_HUMAN	60S ribosomal protein L22	1.048	1.832
85	K2C80_HUMAN	Keratin, type II cytoskeletal 80	1.048	2.146
86	PLST_HUMAN	Plastin-3 OS=Homo sapiens	1.049	-2.115
87	CH60_HUMAN	60 kDa heat shock protein, mitochondrial	1.102	1.783
88	ROA1_HUMAN	Heterogeneous nuclear ribonucleoprotein A1	1.170	1.115
89	HMGB1_HUMAN	High mobility group protein B1	1.170	1.115
90	H2A2B_HUMAN	Histone H2A type 2-B	1.170	1.615
91	MDHM_HUMAN	Malate dehydrogenase, mitochondrial	1.170	2.976
92	DX39B_HUMAN	Spliceosome RNA helicase DDX39B	1.204	1.336
93	HNRPR_HUMAN	Heterogeneous nuclear ribonucleoprotein R	1.204	1.336
94	G3BP1_HUMAN	Ras GTPase-activating protein-binding protein 1	1.204	1.336
95	SRSF4_HUMAN	Serine/arginine-rich splicing factor 4	1.204	1.336
96	LAMP2_HUMAN	Lysosome-associated membrane glycoprotein 2	1.204	1.336
97	TRIM1_HUMAN	Probable E3 ubiquitin-protein ligase MID2	1.204	1.336
98	HS74L_HUMAN	Heat shock 70 kDa protein 4L	1.204	1.336
99	HYOU1_HUMAN	Hypoxia up-regulated protein 1	1.204	1.336
100	HN1_HUMAN	Hematological and neurological expressed 1 protein	1.204	1.723
101	O2T35_HUMAN	Olfactory receptor 2T35	1.204	1.723
102	SMD1_HUMAN	Small nuclear ribonucleoprotein Sm D1	1.204	1.723
103	HNRPL_HUMAN	Heterogeneous nuclear ribonucleoprotein L	1.204	2.494
104	H90B4_HUMAN	Putative heat shock protein HSP 90-beta 4	1.300	1.180
105	SPTN4_HUMAN	Spectrin beta chain, non-erythrocytic 4	1.336	1.115
106	SMD3_HUMAN	Small nuclear ribonucleoprotein Sm D3	1.592	1.336
107	CAZA1_HUMAN	F-actin-capping protein subunit alpha-1	1.592	1.336
108	MT1X_HUMAN	Metallothionein-1X	1.592	1.336
109	TXD17_HUMAN	Thioredoxin domain-containing protein 17	1.592	1.336
110	PTBP1_HUMAN	Polypyrimidine tract-binding protein 1	1.592	1.723
111	STIP1_HUMAN	Stress-induced-phosphoprotein 1	1.592	1.723
112	PHB_HUMAN	Prohibitin	1.592	1.723

113	FKB1A_HUMAN	Peptidyl-prolyl cis-trans isomerase FKBP1A	1.592	1.723
114	TCPW_HUMAN	T-complex protein 1 subunit zeta-2	1.592	2.028
115	NONO_HUMAN	Non-POU domain-containing octamer-binding protein	1.592	2.028
116	CATD_HUMAN	Cathepsin D	1.592	2.846
117	XRCC5_HUMAN	X-ray repair cross-complementing protein 5	1.592	2.846
118	RAB7A_HUMAN	Ras-related protein Rab-7a	1.897	1.336
119	CAN1_HUMAN	Calpain-1 catalytic subunit	1.897	1.336
120	CRIP1_HUMAN	Cysteine-rich protein 1	1.897	1.723
121	RA1L2_HUMAN	Heterogeneous nuclear ribonucleoprotein A1-like 2	1.897	2.028
122	ADT4_HUMAN	ADP/ATP translocase 4	1.897	2.028
123	H2AX_HUMAN	Histone H2AX	1.897	2.994
124	K2C8_HUMAN	Keratin, type II cytoskeletal 8	1.973	2.198
125	AATM_HUMAN	Aspartate aminotransferase, mitochondrial	2.148	1.336
126	PDIA4_HUMAN	Protein disulfide-isomerase A4	2.148	1.723
127	KRT35_HUMAN	Keratin, type I cuticular Ha5	2.148	2.028
128	G6PI_HUMAN	Glucose-6-phosphate isomerase	2.395	1.615
129	K1H1_HUMAN	Keratin, type I cuticular Ha1	2.549	1.336
130	ENOG_HUMAN	Gamma-enolase	2.549	2.028
131	H2B1M_HUMAN	Histone H2B type 1-M	2.549	2.681
132	NUCL_HUMAN	Nucleolin	2.589	3.267
133	G6PD_HUMAN	Glucose-6-phosphate 1-dehydrogenase	3.234	2.028
134	RLA0_HUMAN	60S acidic ribosomal protein P0	3.234	2.494
135	K1C18_HUMAN	Keratin, type I cytoskeletal 18	3.309	3.244
136	FSCN1_HUMAN	Fascin	3.375	1.646
137	K1C17_HUMAN	Keratin, type I cytoskeletal 17	3.519	1.104
138	K2C72_HUMAN	Keratin, type II cytoskeletal 72	3.847	2.028
139	RADI_HUMAN	Radixin	3.984	3.660
140	K1C19_HUMAN	Keratin, type I cytoskeletal 19	4.292	3.363
141	1433S_HUMAN	14-3-3 protein sigma	4.608	1.723

142	ACTG_HUMAN	Actin, cytoplasmic 2	4.913	6.457

Expression levels of these 143 proteins were more than 2-fold higher or lower in pancreatic cancer cells (BxPC-3 and PANC-1) compared to non-cancerous pancreas cells (HPNE).

Table S4. Proteins categorized as extracellular space proteins.

No.	Protein name	fold change (Rsc)	
		BxpC-3	PANC-1
1	Fibronectin	-5.814	-6.532
2	Plasminogen activator inhibitor 1	-4.267	-4.136
3	Putative elongation factor 1-alpha-like 3	-3.878	-3.747
4	Collagen alpha-1(I) chain	-3.347	-3.216
5	Rho GDP-dissociation inhibitor 1	-2.050	-2.768
6	Fibulin-1	-1.941	-1.810
7	Serpin I2	-1.941	-1.810
8	Collagen alpha-3(VI) chain	-1.941	-1.810
9	Carbonyl reductase [NADPH] 3	-1.941	-1.810
10	Gelsolin	-1.833	-1.702
11	Peroxiredoxin-6	-1.817	-1.381
12	Collagen alpha-1(III) chain	-1.554	-1.422
13	Dermcidin	-1.512	-1.686
14	Calreticulin	-1.249	-1.584
15	60 kDa heat shock protein, mitochondrial	1.102	1.783
16	High mobility group protein B1	1.170	1.115
17	Lysosome-associated membrane glycoprotein 2	1.204	1.336
18	Cathepsin D	1.592	2.846
19	Keratin, type I cuticular Ha5	2.148	2.028
20	Glucose-6-phosphate isomerase	2.395	1.615
21	Keratin, type I cuticular Ha1	2.549	1.336
22	Gamma-enolase	2.549	2.028
23	Radixin	3.984	3.660
24	14-3-3 protein sigma	4.608	1.723
25	Actin, cytoplasmic 2	4.913	6.457

Twenty-five proteins that were differentially expressed in pancreatic cancer cells were classified as extracellular space proteins by gene ontology analysis.

Table S5. Clinical and pathological data of patients with pancreatic cacner.

	No.	Age	Gender	pTNM	Albumin (g/dl)	CRP (mg/dl)	CEA (ng/ml)
Pancreatic cancer							
	1	66	F	T4N1M1	3.8	0.089	4.8
	2	68	F	T3N1M1	3.8	0.518	1.0
	3	76	F	T3N0M1	3.2	1.519	4.3
	4	74	M	T3N0M0	3.5	2.382	2.5
	5	76	F	T2N0M1	4.4	0.516	135
	6	85	F	T3N0M0	3.6	0.419	6.3
	7	73	F	T4N0M1	3.7	0.234	2.4
	8	70	M	T4N0M1	3.9	0.208	5.4
	9	61	M	T3N1M1	3.8	3.720	1.6
	10	73	M	T3N1M0	4.4	0.058	3.1
Healthy person							
	1	41	M				
	2	40	F				
	3	32	M				
	4	68	M				
	5	62	F				
	6	34	F				

M, male; F, female.
