# Supplementary figures and images for: Shotgun Label-Free Proteomic Analysis for Identification of a Potential Diagnostic Biomarker for Pancreatic Cancer
Source: Biomedicines. 2025 Oct 27;13(11):2631. doi: 10.3390/biomedicines13112631 (PMC12649963; doi:10.3390/biomedicines13112631)

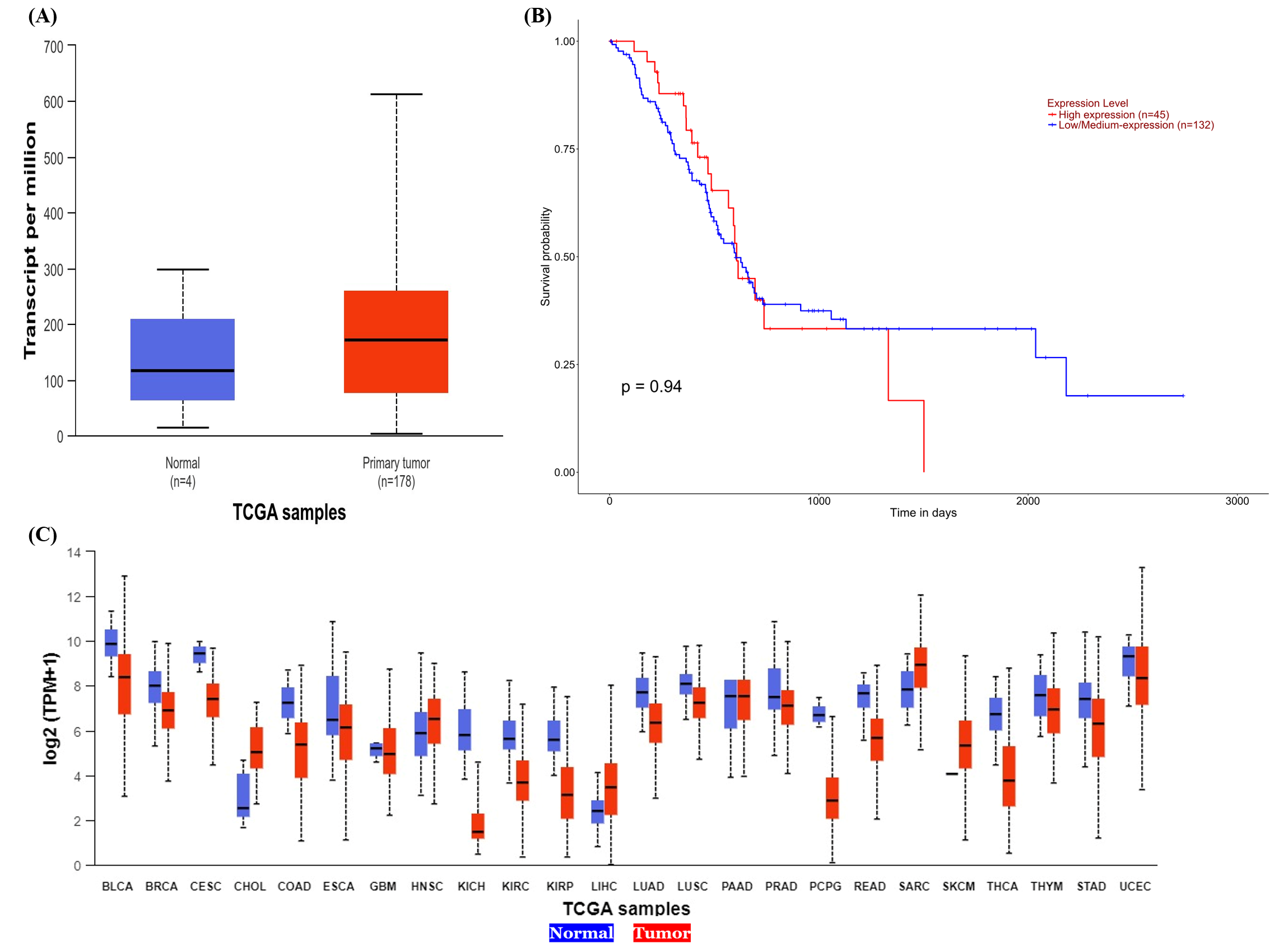

Supplement: Supplementary file 1 [file biomedicines-13-02631-s001.zip › Fig.S2.tif]
